# Genetic Regulation of Virulence and Antibiotic Resistance in *Acinetobacter baumannii*

**DOI:** 10.3390/genes8010012

**Published:** 2016-12-28

**Authors:** Carsten Kröger, Stefani C. Kary, Kristina Schauer, Andrew D. S. Cameron

**Affiliations:** 1 Department of Microbiology, School of Genetics and Microbiology, Moyne Institute of Preventive Medicine, Trinity College Dublin, Dublin 2, Ireland; karys@tcd.ie; 2 Department of Veterinary Science, Faculty of Veterinary Medicine, Ludwig-Maximilians-Universität München, Oberschleißheim 85764, Germany; kristina.schauer@mh.vetmed.uni-muenchen.de; 3 Department of Biology, University of Regina, Regina, SK S4S 042, Canada; andrew.cameron@uregina.ca

**Keywords:** *Acinetobacter baumannii*, virulence gene regulation, two-component systems, multidrug efflux pumps, metal acquisition, antibiotic resistance

## Abstract

Multidrug resistant microorganisms are forecast to become the single biggest challenge to medical care in the 21st century. Over the last decades, members of the genus *Acinetobacter* have emerged as bacterial opportunistic pathogens, in particular as challenging nosocomial pathogens because of the rapid evolution of antimicrobial resistances. Although we lack fundamental biological insight into virulence mechanisms, an increasing number of researchers are working to identify virulence factors and to study antibiotic resistance. Here, we review current knowledge regarding the regulation of virulence genes and antibiotic resistance in *Acinetobacter baumannii*. A survey of the two-component systems AdeRS, BaeSR, GacSA and PmrAB explains how each contributes to antibiotic resistance and virulence gene expression, while BfmRS regulates cell envelope structures important for pathogen persistence. *A. baumannii* uses the transcription factors Fur and Zur to sense iron or zinc depletion and upregulate genes for metal scavenging as a critical survival tool in an animal host. Quorum sensing, nucleoid-associated proteins, and non-classical transcription factors such as AtfA and small regulatory RNAs are discussed in the context of virulence and antibiotic resistance.

## 1. Introduction

Bacterial infections are a leading cause of death worldwide, and the fading age of antibiotics increases risks associated with basic healthcare and surgeries. Moreover, the continuous emergence of multidrug resistant (MDR) microorganisms is a major economic burden, yet the development of new antimicrobials has stalled [1,2]. The most prominent MDR bacterial genera currently evading antimicrobial treatment and spreading worldwide as nosocomial pathogens have been dubbed the ESKAPE organisms [2,3,4]: *Enterococcus faecium*, *Staphylococcus aureus*, *Klebsiella pneumoniae*, *Acinetobacter baumannii*, *Pseudomonas aeruginosa* and *Enterobacter* species. Notably, infections caused by the Gram-negative coccobacillus *Acinetobacter baumannii* are growing in number in hospitals around the world [5,6,7,8,9]. The genus *Acinetobacter* belongs to the class Gamma-proteobacteria, the order Pseudomonadales, and the family Moraxellaceae. Only a few members of this genus have a history of infecting humans, of which *A. baumannii*, *A. pittii* and *A. nosocomialis* are the most clinically relevant [10,11], while many other *Acinetobacter* species are non-pathogenic. Infections of humans are mostly, but not exclusively, hospital-acquired. Community-acquired infection with *A. baumannii* is rare and often occurs in predisposed individuals, mostly in tropical regions [12]. Predisposing factors may include diabetes, chronic lung conditions or post-trauma and adverse lifestyles, such as alcohol abuse and smoking [13].

In the United States, *A. baumannii* is now responsible for more than 10% of nosocomial infections [14]. Critically ill patients, including intensive care unit patients, are at a particularly high risk of infection by *A. baumannii*. The bacterium causes a variety of diseases such as ventilator-associated pneumonia (VAP), bacteraemia, skin and soft tissue infections, endocarditis, urinary tract infections and meningitis [6,10]. The mortality rate in patients is debated, but it can reach up to 35% or even higher [7,15] and likely depends on a patient’s individual condition and the *A. baumannii* strain. Carbapenem-resistant *A. baumannii* (CRAB) strains in particular have emerged as one of the most concerning antibiotic-resistant pathogens [16]. Based on recent estimates, over 50% of *A. baumannii* isolates from intensive care units are carbapenem-resistant, and therapy of CRAB infections is estimated to cost global healthcare systems in excess of 742 million US Dollars annually [17]. Of particular concern is the isolation of pan-resistant *A. baumannii* strains across the world, illustrating the ongoing emergence of increasingly dangerous isolates [18,19,20,21,22,23].

To successfully combat the spread of MDR bacterial pathogens, we must develop sufficient biological understanding to engineer new and effective interventions. Despite the growing clinical importance of pathogenic strains of *A. baumannii*, the scientific community has only begun to understand the fundamentals of *A. baumannii* infection biology. A number of studies have identified virulence factors, from extracellular matrices and biofilm formation to drug efflux pumps. For example, the impact of cell surface-associated virulence factors and secretion systems on cell–host interaction has been reviewed recently [24].

A next step is to understand the mechanisms that control expression of virulence factors, because bacteria must carefully control gene expression to ensure correct spatiotemporal production. Regulated expression integrates virulence factors into cellular physiology and reflects how the pathogen is sensing and interacting with host-associated environments and other niches. To combat antibiotic resistance and develop knowledge-based interventions, detailed understanding of the resistance mechanisms and the regulation of genes conferring drug resistance are required. In this review, we provide an overview of current knowledge of the protein and RNA transcription factors that control virulence gene expression and antibiotic resistance in *A. baumannii*, followed by a brief discussion of research horizons that promise important insights into *A. baumannii* virulence.

## 2. Two-Component Systems

Two-component systems (TCS) are ubiquitous systems of signal transduction in bacteria [25]. A typical TCS is composed of a sensor kinase embedded in the cytoplasmic membrane that is able to sense and respond to extracellular and/or intracellular features, like osmotic pressure or pH [26]. When triggered by the environmental or physiological stimulus, the sensor kinase relays this signal by phosphorylating a cognate response regulator. The response regulator is a transcription factor that undergoes a conformational change upon phosphorylation that facilitates (or in some cases hinders) DNA-binding. Using adenosine triphosphate (ATP), the sensor kinases autophosphorylates at a histidine residue which can be transferred onto an aspartic acid residue of the response regulator, usually leading to a transcriptional response (activation and/or repression). When the stimulus ceases, the phosphorelay system runs in reverse wherein the sensor kinase dephosphorylates the response regulator and reverses the transcriptional response.

**Figure 1:**
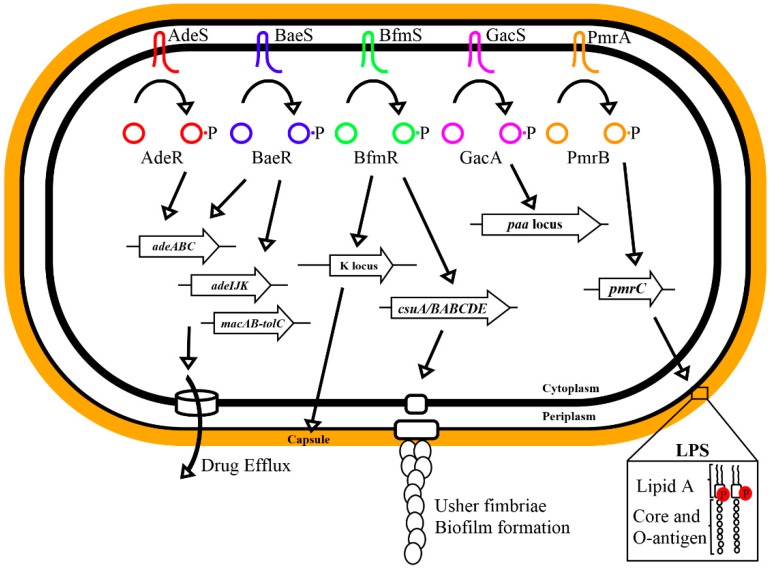
Overview of the impact of five *Acinetobacter baumannii* two-component systems on gene expression of selected genomic loci. LPS: lipopolysaccharide.

Although TCS can be highly conserved between species, the regulons they control can differ even among closely related species [27,28]. Also emerging is an appreciation that both the phosphorylated and unphosphorylated forms of the response regulator can control different regulons. An example is the SsrB protein in *Salmonella*, which activates virulence genes as phosphorylated form and biofilm formation in an unphosphorylated state [29,30]. In *A. baumannii* AB5075-UW, 10 sensor kinases and 17 response regulators are annotated [31]. This is an average number of response regulators for pathogenic bacteria, but lower than the average of 40 in *Escherichia coli* and around 90 in the more closely related *Pseudomonas* [32]. The excess of response regulators highlights the potential for cross-talk among TCS. Five TCS have been studied in *Acinetobacter* (Table 1), and a simple schematic of the regulators and their virulence gene targets is provided in Figure 1. Of these, four TCS are conserved in 15 sequenced *A. baumannii* and one *A. baylyi* genomes provided at the Prokaryotic Genome Analysis Tool webserver (http://tools.uwgenomics.org/pgat/) [33], whereas *adeRS* is missing from *A. baumannii* SDF and *A. baylyi* ADP1.

### 2.1. AdeRS

The *adeRS* TCS is the best characterized regulatory system in *A. baumannii.* Most notably, it controls the expression of the RND-type efflux pump AdeABC [34,35]. Deletion of *adeR* or *adeS* led to increased susceptibility towards aminoglycosides [35]. Many studies have connected antibiotic resistance to increased production of AdeABC, e.g., resistance to tigecycline, fluoroquinolones, aminoglycosides and others [36,37]. Decreased susceptibility was reported to be caused by mutations in *adeRS* which led to overexpression of *adeABC* [38,39,40,41]. Moreover, *adeRS* shows a high level of sequence variation in clinical isolates which could explain the differences in tigecycline resistance [42]. The regulation of *adeABC* is complex and not only controlled by AdeRS, as overexpression of the efflux pump was also observed in a strain lacking insertion mutations in *adeRS* that cause overexpression of the system [43]. The overexpression of AdeABC led to increased virulence in a pulmonary infection model, which suggests that increased levels of AdeABC are beneficial to both antibiotic resistance and in vivo fitness, and can explain why *adeRS* mutations are frequently observed in clinical isolates [43,44,45,46]. However, the AdeRS regulon includes many other genes that contribute to virulence as recently reported using RNA-seq [47]. It was found that AdeRS directly or indirectly regulates 579 genes, most notably those involved in the expression of efflux pumps, biofilm formation and virulence in a *Galleria mellonella* larvae infection model. Intriguingly, some outcomes of the AdeRS deletion appeared to be strain specific. Deletion of *adeRS* in *A. baumannii* AYE resulted in loss of biofilm formation in an ex vivo porcine vaginal mucosa model and a reduced host epithelial cell toxicity, but not in *A. baumannii* S1 [47]. Strain to strain variation in a key feature such as antimicrobial resistance highlights the difficulty of extrapolating a significant biological finding between strains [42,48]. This diversity among *A. baumannii* strains makes treatment even more challenging [49].

### 2.2. BaeSR

The *baeSR* TCS in *Acinetobacter* spp. was so named because the nucleotide sequence indicated high similarity to *baeSR* in *E. coli* and to *Salmonella enterica* serovar Typhimurium [50]. Among other genes, BaeSR controls drug efflux pumps in *E. coli*, which suggested a similar function for *A. baumannii*. Indeed, deletion of *baeSR* led to significantly reduced expression of the major efflux pumps AdeABC, AdeIJK, and MacAB-TolC, resulting in increased susceptibility to tigecycline [50,51]. The regulation of the three efflux pumps is proposed to be indirect, though, as purified BaeR-His did not bind the promoters of *adeA*, *adeI* or *macA* in electrophoretic mobility shift assays. However, it was unclear whether the phosphorylation state of purified BaeR-His was appropriate for DNA binding, or whether the histidine tag might interfere with DNA binding. As the regulons of AdeRS and BaeSR overlap, this could also mean that BaeSR functions through cross-talk with AdeRS. The expression of *baeSR* increased upon growth in elevated sucrose levels, suggesting that BaeS responds to changes in osmotic pressure [50]. In addition, tannic acid which showed potential as an antibiotic adjuvant [52,53], increased gene expression of the three efflux pumps mentioned above [51].

### 2.3. BfmRS

The ability to form biofilms on abiotic surfaces allows *A. baumannii* to resist adverse conditions, such as desiccation or detergents. Protein filaments called pili are required for attachment to surfaces. In *A. baumannii*, the best studied pili involved in biofilm formation are produced by the *csuA/BABCDE* usher-chaperone assembly system, which is regulated by the TCS termed *bfmRS* [54,55]. Deletion of only the response regulator *bfmR* resulted in complete loss of biofilm formation on plastic, while a *bfmS* sensor kinase mutant retained some ability to from biofilm, suggesting that BfmR can be activated by alternate sensor kinases [54]. Whether *csuA/BABCDE* is required for attachment to host tissues is not fully resolved. *csuA/BABCDE* is not involved in adherence to bronchial epithelial cells [56], but deletion of the sensor kinase *bfmS* led a loss of adhesion to A549 human alveolar epithelial cells [57]. However, adhesion to A549 could be independent of *csuA/BABCDE*, if BfmRS controls the regulation of other adhesion factors.

BfmRS both enhances and represses antibiotic resistance. Deletion of *bfmS* led to an increased minimum inhibitory concentration (MIC) of ciprofloxacin and a decreased MIC of imipenem, while deletion of *bfmR* increased the MIC of meropenem and colistin [58]. Moreover, outer membrane proteins, such as the major virulence factor OmpA, were increasingly released into the supernatant, indicating that cellular integrity is compromised. Indeed, BfmRS regulates genes that maintain cellular morphology and control cell division—the direct targets are however unknown [54]. Recently, BfmRS was shown to control gene expression of the K locus, a major virulence determinant in *A. baumannii* ATCC17978, which harbors genes for exopolysaccharide (e.g., capsule) production [59]. Surprisingly, capsule synthesis was not affected in deletions of *bfmR* in two clinical isolates that showed reduced growth/survival in human ascites’ fluid and serum [58]. These diverse phenotypes highlight the multifaceted nature of the BfmRS regulon. Unfortunately, we lack a detailed understanding of the regulon in biofilm-inducing conditions, in an infection model, or a description of strain-specific differences in regulon membership. As BfmR was recently suggested as a candidate drug target, deeper knowledge of the regulon of the BfmRS TCS is therefore of particular interest [58].

### 2.4. GacSA

The GacS sensor kinase was identified through its mutagenesis in a transposon screen of *A. baumannii* ATCC19606; the mutant was unable to use citrate as a sole carbon source [60] and to kill the human fungal pathogen *Candida albicans* [61]. Today, the *gacSA* TCS is established as a global regulator in *A. baumannii* controlling the expression of 674 genes including virulence genes, biofilm and pili formation, resistance against human serum, motility, and metabolism of aromatic compounds [62,63]. Deletion of GacS led to an avirulent phenotype in a mouse septicaemia model and greatly reduced bacterial load in spleen, kidney and liver 12 hours after infection, highlighting the importance of GacS during systemic infection. Intriguingly, GacS controls a catabolic pathway of aromatic compounds (termed *paa*, phenylactetic acid pathway) that was shown to be important for full virulence. Genome comparisons revealed that the *A. baumannii paa* operon is similar to the well-studied *paa* system in *E. coli* [62,64]. The aromatic compounds phenylacetate and phenylalanine are converted into acetyl- and succinyl-CoA and fed into the tricarboxylic acid cycle. Surprisingly, inhibition of the *paa* pathway led to increased killing of *A. baumannii* in a zebrafish infection model. The metabolic by-product phenylacetate accumulates in the *gacS* mutant and represents a potent chemoattractant for neutrophils, thus GacS is involved in innate immune evasion by controlling the expression of the *paa* locus [63]. The GacSA TCS is not a contiguous operon as each gene is located far apart on the chromosome, unlike the AdeRS, BaeSR, PmrAB and BfmRS TCS in *A. baumannii*. Whereas GacS is alone in a transcriptional unit, the response regulator GacA does lie adjacent to another sensor kinase of unknown function (A1S_0235 in *A. baumannii* ATCC17978), suggesting that GacS has co-opted a response regulator to create cross-talk between two TCS.

### 2.5. PmrAB

In response to the increasing drug resistance of *A. baumannii*, including resistance to fluoroquinolones and carbapenems, the cationic peptide antibiotic colistin (polymyxin E) has become a last line of defence to treat infections with MDR *A. baumannii* strains [74,75]. However, colistin-resistant strains have emerged and one mechanism of resistance is mediated by mutations in the TCS encoded by *pmrAB* (polymyxin resistance) [76,77]. Indeed, PmrAB has been implicated in polymyxin B and colistin resistance in several other species, such as *Pseudomonas aeruginosa, Klebsiella pneumoniae* and *Salmonella enterica* [78,79,80,81]. In addition, the *pmrAB* TCS has been shown to regulate genes involved in lipopolysaccharide modification [80,82]. Colistin resistance in *A. baumannii* is conferred by mutations in *pmrAB* that lead to overexpression of the response regulator PmrA and/or the sensor kinase PmrB, which suggests that differential expression of members of the *pmrAB* regulon are responsible for the resistant phenotype. However, the complete regulon controlled by PmrAB in *A. baumannii* has not been investigated in detail.

Modifications of lipopolysaccharides (LPS) in the cell envelope are recognized mechanisms that cause polymyxin resistance. For instance, in *Salmonella*, addition of 4-amino-4-deoxy-l-arabinose or phosphoethanolamine to lipid A reduces the overall negative surface charge of the bacterial cell and thereby decreases electrostatic interactions with cationic peptides [83,84,85,86,87]. As well, changes in PmrB levels in colistin-resistant *A. baumannii* strains led to modification of lipid A with phosphoethanolamine [65,88]. Conversely, complete loss of LPS increases colistin resistance, but it is yet to be determined whether *pmrAB* is directly involved in this resistance [89]. Slightly acidic pH (pH 5.5), ferric iron (Fe^3+^) or low magnesium levels could be the signals sensed by the sensor kinase PmrB, as these environmental conditions increase the MIC for colistin [76,88,90]. Acquiring colistin resistance through mutations in *pmrAB* can lead to decreased bacterial fitness in in vitro growth experiments and in in vivo infection models [91,92,93,94]. For example, colistin-resistant clinical isolates showed impaired virulence in a rat model of pneumonia [95] and in intraperitoneally infected mice that were attributed to mutations in PmrAB [91]. In addition to conferring resistance to polymyxins, PmrAB might control the expression of a set of virulence factors or the changes of lipid A modification could directly account for the altered virulence phenotypes [88].

## 3. Regulation of Iron and Zinc Acquisition

The acquisition of metals, such as iron, zinc or manganese, is essential for all living organisms, because they are key to many important physiological processes. Depleting bacterial pathogens of essential metals is a host strategy to limit pathogen expansion—a strategy termed nutritional immunity [96,97,98]. The host expresses a set of proteins, such as haemoglobin, transferrin, lactoferrin or calprotectin that sequester iron, zinc and manganese making them less available for an invading pathogen. To subvert the host defence mechanisms, bacterial pathogens have evolved strategies to counter metal limitation by expressing efficient metal uptake systems and secrete molecules that bind metals in the environment and that may subsequently be taken up again. In *A. baumannii*, the regulation of the systems controlling iron and zinc acquisition have been studied and are reviewed below.

### 3.1. Fur—the Ferric Uptake Regulator

Iron is required for many cellular processes, for instance for DNA precursor synthesis or as cofactors in redox enzymes. To compete for iron with the host, bacteria have evolved intricate iron acquisition mechanisms. One mechanism to secure free iron is to secrete iron-chelating molecules (siderophores) which may later be taken up again from the environment or to scavenge iron bound to host transferrin and lactoferrin [99]. In bacteria, siderophore-encoding genes are often located in gene clusters. In *A. baumannii*, genomic comparisons have identified five siderophore gene clusters, however, *A. baumannii* isolates may possess different siderophore repertoires [66,100]. One well-characterized siderophore is termed acinetobactin [99,101], which is structurally and functionally related to anguibactin from *Vibrio anguillarum* [102]. Acinetobactin-facilitated iron acquisition has been reported to be an important virulence factor in several *A. baumannii* infection models [103]. Upon inactivation of the acinetobactin-mediated iron uptake and utilization system, reduced apoptosis in *A. baumannii* infected alveolar epithelial cells A549 was reported and decreased virulence in *Galleria mellonella* larvae infections and a mouse sepsis model was observed [103]. In many Gram-negative bacteria including *A. baumannii*, iron acquisition-related genes are controlled by the ferric uptake regulator (Fur). The Fur protein is a transcriptional repressor that binds a 19 bp AT-rich consensus sequence, called the “Fur box” when containing ferrous iron (Fe^2+^) [66], and the Fur amino acid sequence in *A. baumannii* is 63% identical with the well-studied Fur protein in *E. coli* [104]. In response to iron limitation in vitro, 1207 *A. baumannii* ATCC17978 genes showed differential gene expression [66]. The siderophore clusters 1 and 2 and the acinetobactin cluster were strongly upregulated clusters and they possess candidate Fur-binding sites suggesting that Fur is directly involved in expression of siderophores. A second Fur-like protein (ABEYE1889) has been discovered in *A. baumannii*, however, it appears to be restricted to the *A. baumannii* AYE strain so far [66]. Functional characterization of ABEYE1889 is lacking, however, it might add complexity to the control of iron homeostasis. Fur has also been suggested to be involved in regulating the *tonB-exbB-exbD* system which is energizing transport processes across the outer membrane, for example of iron-siderophore complexes [105,106]. In *A. baumannii* ATCC19606, three *tonB* genes have been identified and only the expression of *tonB_3_* responds to iron levels and is predicted to be Fur-regulated. The *tonB-exbB-exbD* might play a role in *A. baumannii* infections, as TonB proteins were required for full virulence in *Galleria mellonella* larvae infections [105].

### 3.2. Zur—the Zinc Uptake Regulator

In bacteria, zinc is required for the action of metalloproteins, such as metalloproteases [107]. In response to zinc limitation, *A. baumannii* upregulates an inner membrane ABC transporter encoded by the *znuABC* genes to increase zinc uptake [67]. The regulation of zinc-associated genes is controlled by Zur (zinc uptake regulator), a zinc-binding regulatory protein and a member of the Fur protein family [67,68]. Like Fur, Zur functions as a transcriptional repressor binding to DNA at 19 bp consensus sequence in a zinc-dependent manner. De-repression of Zur-controlled genes occurs in zinc-depleted conditions, when the Zur apoprotein can no longer bind to DNA. In an *A. baumannii* transcriptomic study, deletion of *zur* resulted in differential expression of 144 genes, however, it is unclear how many are directly regulated by Zur. Among the differentially expressed genes were 11 (seven upregulated and four downregulated in the *zur* mutant strain) transcription factors suggesting significant cross-talk with other regulatory systems. Zinc acquisition is crucial for *A. baumannii* virulence. In a mouse pneumonia model, Zur was shown to be required for dissemination to the liver and this effect was abolished in calprotectin-deficient mice [67,68].

Considered together, this illustrates the importance of precise regulation of metal acquisition systems and homeostasis for *A. baumannii* during infection. Thus, antimicrobials targeting these systems might prove to be particularly promising. For instance, the iron mimetic gallium nitrate (Ga(NO_3_)) inhibited growth of 58 *A. baumannii* strains in defined medium and in human serum and increased the survival rate of *A. baumannii*-infected *Galleria mellonella* larvae [108]. In addition, gallium nitrate dispersed *A. baumannii* biofilms produced in complement-free human serum [109]. Gallium competes with iron for bacterial iron acquisition systems and iron-binding proteins and may inhibit their function, because unlike Fe^3+^, Ga^3+^ cannot be reduced under physiological conditions [110,111]. Thus, gallium-based compounds may be included in effective antibacterial treatment strategies.

## 4. Biofilm Formation and Quorum Sensing

Biofilm formation by pathogenic bacteria is a challenge in healthcare because biofilms make bacteria more resistant to antibiotics, reduce the effectiveness of decontamination efforts, and increase persistence of bacteria on indwelling devices. Multiple regulatory pathways have been implicated in biofilm formation by *A. baumannii*, including the surprising finding that biofilm formation on polystyrene surfaces requires BfmR (described above), but not BfmS [54]. Several studies have linked biofilm formation by *A. baumannii* to quorum sensing. *A. baumannii* encodes *abaI* and *abaR*, homologs of *Vibrio*’s archetypal quorum sensing genes *luxI* and *luxR*, respectively [112,113]. The enzyme AbaI produces *N*-acyl-homoserine lactone (AHL), a diffusible molecule that functions as an autoinducer by binding the AHL-responsive transcription factor AbaR [112]. Deleting *abaI* reduces biofilm formation in *A. baumannii* [69,112]. Surveys of clinical isolates indicate that about half can form biofilms in laboratory conditions, yet only a subset of biofilm-forming strains produce AHL [69,70]. Bacteria transition from free-living to biofilm physiology by producing adherent proteins and extracellular matrices that reinforce biofilm structures; for example, in the well-characterized model *Pseudomonas*, genes for biofilm formation are regulated by quorum sensing systems [114]. Thus, it is reasonable to hypothesize that AbaR activates biofilm gene expression in *A. baumannii* when AHLs rise to autoinducing concentrations. Curiously, some *A. baumannii* strains only encode an orphan *abaI* that appears to lack the cognate response regulator *abaR* [70]. This is counter to genome-wide analyses which indicate that *luxR* homologs usually outnumber *luxI* homologs [115], and that some species have a *luxR* homolog for sensing AHL signals but lack *luxI* and so do not contribute to signalling [116]. Could the unusual *A. baumannii* strains represent a special case of organisms that generate AHLs but do not sense them? In other words, might these *A. baumannii* strains generate AHLs for interspecies signalling to manipulate gene expression in neighbouring strains? Alternatively, in the absence of AbaR, AbaI may cross-talk with other quorum sensing systems in the same cell.

## 5. Other Regulatory Proteins

### 5.1. AtfA

The acidic transcription factor A (AtfA) in *Acinetobacter* spp. is a small protein (67–68 amino acids) that was first functionally described in *A. baylyi* ADP1 [71]. The gene has been unfortunately termed *atfA*, which is a name shared with the wax ester synthase/acyl-CoA:diacylglycerol acetyltransferase enzyme in *A. baylyi* ADP1 [117,118]. Deletion of *atfA* transcription factor resulted in gene expression changes of over 500 genes including several virulence-associated traits. Massive phenotypic changes were observed in *atfA* deletions in *A. baylyi* ADP1 and *A. baumannii* ATCC19606, including cell enlargement, loss of biofilm formation, reduced twitching motility and increased susceptibility to antibiotics and ethanol. Differences in response to ethanol might alter the expression of ethanol-induced virulence factors, such as phospholipase C [119]. Mechanistically, AtfA does not seem to be a classical transcription factor, but was shown to bind directly to RNA polymerase in a 1:1 ratio. This displaced DNA from RNA polymerase core complexes, but did not displace DNA from the holoenzyme containing a sigma factor [71]. The location of *atfA* adjacent to the gene encoding the major sigma factor 70 could be significant for the co-regulation of this transcription factor with core transcriptional machinery. Thus, the extent of gene expression and phenotypic changes and the mechanistic insight into AtfA action indicate that AtfA is a global regulator. AtfA is conserved in the gamma-proteobacteria *Moraxella*, *Pseudomonas*, *Legionella*, *Vibrio* and *Acinetobacter*, but absent in Enterobacteriaceae [71].

### 5.2. Nucleoid-Associated Protein H-NS

Nucleoid-associated proteins and DNA supercoiling shape the bacterial nucleoid [120,121]. By virtue of their high abundance, binding across the bacterial chromosome, and the ability to constrain regions of DNA supercoiling, nucleoid-associated proteins affect the expression of many hundreds of genes in model organisms like *E. coli* and *Salmonella* [122]. *Acinetobacter* sp. encode an H-NS homolog that can complement an *E. coli hns* deletion mutant [72]. Deletion of the *A. baumannii* ATCC 17978 *hns* homolog (A1S_0268) caused a hyper-motile phenotype along with enhanced adherence to human pneumocytes and enhanced virulence in a nematode infection assay [73]. Ninety-one genes were upregulated in the *hns* mutant, including the genes encoding the virulence-associated adhesin *ata* (A1S_1032), a type-VI secretion system (A1S_1292-1311), and genes for type I pili. The most highly upregulated genes had AT-rich promoter regions, consistent with H-NS affinity for AT-rich DNA in the well-studied *E. coli* and *Salmonella* systems. A role for *A. baumannii* H-NS in xenogeneic silencing was reported as several genomic islands were found to be repressed by H-NS [73]. To our knowledge, gene regulatory effects of DNA supercoiling have not been studied in *Acinetobacter*.

## 6. Global Studies Identifying Important Regulators

Random global mutagenesis studies in bacteria have been instrumental to studying gene function. About a decade ago, classical genetic screenings using mobile genetic elements, such as transposons, have been paired with next-generation sequencing to allow high throughput identification and quantification of transposon insertions to assess the contribution of transposon-disrupted genes to bacterial fitness [123,124]. Among many other studies, transposon-insertion sequencing allowed the identification of *Salmonella* genes involved in colonization of farm animals [125], revealed novel genotype–phenotype relationships in *Streptococcus pneumoniae* [126] and detected essential genes in Rhizobiaceae [127]. Transposon-insertion sequencing in a *A. baumannii* ATCC17978 pneumonia model identified seven regulatory proteins that contribute to lung persistence (Table 2, [128]). Among them are the aforementioned response regulators GacA and BfmR, as well as the BfmS sensor kinase. Integration host factor (IHF), a homolog of the *E. coli* nucleoid-associated protein IHF, was shown to be required for virulence, however, H-NS was not. *Acinetobacter junii* and *E. coli* IHF bind similar DNA sites and both can repress transcription [129], but little else is known about *Acinetobacter* IHF function. Other predicted regulators required for persistence in the mouse lung are two LysR-type regulators (A1S_2122, A1S_2537, Table 2) and the putative high affinity phosphate uptake regulator *phoU* (Table 2), none of which have been studied in detail. Prediction of gene function suggested involvement of A1S_2122 in methionine synthesis [128]. The gene encoding A1S_2537 lies downstream of a number of genes predicted to mediate sulfate transport. Thus, both regulators might be involved in metabolism of sulfur-containing molecules. More recently, Gebhardt et al. performed a transposon-screen in *A. baumannii* strain AB5075 showing that virulence and resistance genes appear to be co-regulated in response to antibiotic and environmental stress [130]. In the same study, 17 (putative) regulatory proteins were identified that were required for growth in *Galleria mellonella* larvae (Table 3), suggesting that strains possessing increased resistance to antibiotics might simultaneously possess improved virulence properties.

The large number of important, yet functionally uncharacterized virulence gene regulators (Table 2 and Table 3) highlight the need to study their regulons to obtain a more complete picture of the regulatory networks governing virulence gene expression. These approaches need to be extended to study antibiotic resistance genes and their regulation to reveal novel potential target for drug intervention. Novel global genomic approaches, such as CRISPR interference (CRISPRi), promise to accelerate the understanding of gene function and regulation [124,131].

## 7. Do Small Regulatory RNAs Have a Role in *A. baumannii* Virulence or Antibiotic Resistance?

Small RNAs (sRNAs) are now fully established as a class of gene regulators of various cellular processes in bacteria [132,133,134,135]. Best understood are expression patterns and regulatory mechanisms employed by small non-coding RNAs in Enterobacteriaceae such as *E. coli* and *Salmonella* [136,137,138,139,140]. So far, we lack information as to whether sRNA-mediated regulation in *A. baumannii* is analogous to these well-studied systems. Bioinformatic analysis of the *A. baumannii* ATCC17978 genome identified 31 putative small RNA candidates, of which three were confirmed by Northern blotting using strain ATCC15308 [141]. In the multidrug resistant *A. baumannii* strain AB5075, 78 small RNAs were identified using RNA-sequencing [142]. However, we lack insight as to whether these sRNA candidates possess any physiological function or how they might function mechanistically.

In Enterobacteriaceae, one function of the RNA-binding protein Hfq is to facilitate the interaction of sRNA and their cognate mRNA targets [143]. Yet in *Acinetobacter*’s closer relative, *Pseudomonas aeruginosa*, the role of Hfq in sRNA-mediated regulation is less clear and does not seem to follow the same model as in Enterobacteriaceae [144,145]. In *Acinetobacter* spp., Hfq has only been studied in the avirulent *A. baylyi*. Hfq possesses an unusually long, glycine-rich C-terminus, which is a unique feature of *Acinetobacter* spp. Hfq. This may impact how and where RNAs bind to Hfq, as well as how Hfq interacts with other cellular players [146]. In other bacterial pathogens, deletions in RNA binding proteins such as Hfq or CsrA often results in avirulent phenotypes [147,148,149,150], however, the role of Hfq in *A. baumannii* virulence or antibiotic resistance remains unknown. Only the sRNA Aar has been studied as a regulatory RNA in *A. baylyi*. Aar, which is conserved in *A. baumannii* [142,151], was suggested to play a role in amino acid metabolism of *A. baylyi*, but mechanistic details remain unknown [151]. Several Hfq-dependent sRNAs have been described to control iron homeostasis in other Gram-negative bacteria [152,153] and there might be a connection of sRNA-mediated gene regulation and iron homeostasis in *A. baumannii*, because a proteomic study showed that *A. baumannii* Hfq protein levels decreased in iron-limiting conditions [154]. Moreover, Hfq protein and Hfq-dependent sRNAs have been identified to play a role in antibiotic resistance, as small RNAs control the expression of many cellular targets of antibiotic action [155,156,157]. In Enterobacteriaceae, outer membrane homeostasis of major porins OmpA, OmpC, OmpD and OmpF is controlled by the concerted action of several small RNAs [158,159,160,161,162,163]. In addition, the outer membrane component of the RND superfamily multidrug transporter TolC is regulated by the sRNA SdsR in *E. coli* [164]. Considering the prominent role of OmpA in *A. baumannii* virulence and drug resistance [165,166,167], it will be interesting to investigate whether a similar sRNA-mediated control of *A. baumannii* OmpA and other outer membrane proteins exists.

Considered together, it will be important to elucidate the regulons and regulatory mechanisms of sRNA along with identification and characterisation of RNA binding proteins in *Acinetobacter*—especially during infection and upon exposure to antibiotics.

## 8. Conclusions and Outlook

Bacterial pathogens must adapt quickly to changing environments, for example, during host entry or in harsh environmental conditions, and modulate their gene expression programs accordingly. This is achieved by multiple regulatory systems that govern correct spatiotemporal gene expression. In the bacterial pathogen *A. baumannii*, an increasing number of virulence genes have been identified over the last few years, however, their gene regulation remains largely unknown. So far, very few regulons have been defined and we lack data for all *A. baumannii* transcription factors whether regulatory effects are direct or indirect. This highlights the need to use and combine global approaches such as DNA binding studies using chromatin-immunoprecipitation (ChIP) sequencing, transposon-insertion sequencing, CRISPR interference and RNA-sequencing to define direct and indirect effects of regulatory proteins in *A. baumannii* and to identify regulators that control virulence gene expression and antibiotic resistance. In addition, the extent and mechanisms of RNA-mediated gene regulation remain completely unknown. To improve the ability to study gene regulation in detail, the range of basic molecular tools, such as reporter plasmids, needs to be expanded, however, this can be hampered by the multidrug resistant genotypes of clinical isolates and the lack of detailed knowledge about restriction modification systems in *A. baumannii*. Moreover, to make generalizing statements about *A. baumannii* virulence and antibiotic resistance as well as their genetic regulation is complicated by the significant strain-to-strain differences. Thus, we require multiple efforts to understand how genes are regulated in this important opportunistic pathogen that could inform the development of knowledge-based interventions.

## Figures and Tables

**Table 1:** Best characterized regulatory systems in *Acinetobacter baumannii*.

Gene Name	Type	Function and Phenotype	Relevant References
*adeRS*	Two-component system	Directly or indirectly controls 579 genes. Regulates the expression of the AdeABC drug efflux pump. Required for biofilm formation biofilm.	[35,47]
*bfmRS*	Two-component system	Controls the expression of the K locus (exopolysaccharide production/capsule). Regulates expression of CsuA/BABCDE chaperone-usher secretion system, biofilm formation on abiotic surfaces.	[54,58,59]
*gacSA*	Two-component system	Directly or indirectly controls 674 genes, including virulence genes, biofilm and pili formation, resistance against human serum, motility, and metabolism of aromatic compounds (*paa* locus).	[60,62,63]
*baeSR*	Two-component system	Deletion results in reduced expression of AdeABC, AdeIJK, MacAB-TolC drug efflux pumps and increases susceptibility to tannic acid.	[50,51]
*pmrAB*	Two-component system	Mutations in *pmrAB* can confer resistance to colistin.	[65]
*fur*	Transcription factor	Transcriptional repressor of genes involved in iron homeostasis.	[66]
*zur*	Transcription factor	Transcriptional repressor of genes involved in zinc homeostasis.	[67,68]
*abaR*	Transcription factor	AHL-responsive transcription factor involved in quorum sensing and biofilm formation.	[69,70]
*atfA*	RNA polymerase binding protein	Deletion of *atfA* results in cell enlargement, loss of biofilm formation and increased susceptibility to antibiotics and ethanol.	[71]
*hns*	Nucleoid-associated protein	Deletion results in a hyper-motile phenotype, enhanced adherence to human pneumocytes and enhanced virulence in a nematode infection assay.	[72,73]

**Table 2:** Regulators required for persistence in the mouse lung (ATCC17978; [128]).

Gene Name	Gene Identifier			Product
	ATCC17978	ATCC17978-mff	AB5075-UW	
*gacA*	A1S_0236	ACX60_RS16900	ABUW_RS17720	Two-component response regulator
*phoU*	A1S_0256	ACX60_RS16800	ABUW_RS17620	High affinity phosphate uptake transcriptional repressor
*ihfA*	A1S_0603	ACX60_RS15110	ABUW_RS15930	Integration host factor subunit alpha
*bfmR*	A1S_0748	ACX60_RS14635	ABUW_RS15450	Two-component response regulator
*bfmS*	A1S_0749	ACX60_RS14630	ABUW_RS15445	Two-component sensor histidine kinase
-	A1S_2122	ACX60_RS06950	ABUW_RS07810	LysR family transcriptional regulator
-	A1S_2537	ACX60_RS04880	ABUW_RS04985	LysR family transcriptional regulator

**Table 3:** Regulators required for growth in *Galleria mellonella* (AB5075; [130]).

Gene Name	Gene Identifier			Product
	ATCC17978	ATCC17978-mff	AB5075-UW	
-	A1S_2082	ACX60_RS11775	ABUW_RS08030	TetR family transcriptional regulator
-	A1S_2064	ACX60_RS07270	ABUW_RS08145	LysR family transcriptional regulator
-	A1S_2042	ACX60_RS07365	ABUW_RS08240	TetR family transcriptional regulator
-	A1S_1958	ACX60_RS08015	ABUW_RS08550	AsnC family transcriptional regulator
-	A1S_1948	ACX60_RS08070	ABUW_RS08610	MarR family transcriptional regulator
-	A1S_1874	ACX60_RS08460	ABUW_RS09000	LysR family transcriptional regulator
-	not present	not present	ABUW_RS09560	LysR family transcriptional regulator
-	not present	not present	ABUW_RS10070	Fur family transcriptional regulator
*alkR*	A1S_1640	ACX60_RS09755	ABUW_RS10675	AraC family transcriptional regulator
*-*	A1S_1578	ACX60_RS10205	ABUW_RS10870	AraC family transcriptional regulator
*arsR*	A1S_1453	ACX60_RS10865	ABUW_RS11530	ArsR family transcriptional regulator
*-*	A1S_1350	ACX60_RS11395	ABUW_RS12250	TetR family transcriptional regulator
*-*	A1S_1330	ACX60_RS11505	ABUW_RS12365	AraC family transcriptional regulator
*soxR*	A1S_1320	ACX60_RS11550	ABUW_RS12410	Redox-sensitive transcriptional activator
*-*	A1S_0768	ACX60_RS14535	ABUW_RS15350	LysR family transcriptional regulator
*bfmS*	A1S_0749	ACX60_RS14630	ABUW_RS15445	Two-component sensor histidine kinase
-	A1S_0621	ACX60_RS15010	ABUW_RS15830	Putative two-component response regulator
